# The Persistent Approach to Diagnose Infectious Hepatic Cysts in a Patient With Recurrent Fever: A Case Report

**DOI:** 10.7759/cureus.21137

**Published:** 2022-01-11

**Authors:** Hirotaka Ikeda, Ryuichi Ohta, Nozomi Nishikura, Yoshinori Ryu, Chiaki Sano

**Affiliations:** 1 Community Care, Unnan City Hospital, Unnan, JPN; 2 Communiy Care, Unnan City Hospital, Unnan, JPN; 3 Community Medicine Management, Shimane University Faculty of Medicine, Izumo, JPN

**Keywords:** autosomal recessive polycystic kidney, comprehensive physical exam, polycystic liver, rural hospitals, recurrent fever, infectious hepatic cyst

## Abstract

Diagnosing infectious hepatic cysts (IHCs) can be challenging. Moreover, patients with IHCs may present with various symptoms. Diagnosis of IHCs can be even more difficult in patients with multiple liver cysts. For appropriate diagnosis, the detection of infectious sections in the liver is essential. However, diagnosing and determining definite treatments for patients with IHCs can be particularly challenging when they have polycystic liver disease. We present a case of a 70-year-old man who visited a rural community hospital with a primary complaint of recurrent fever and pain in the right upper quadrant. Based on his clinical history, physical examination findings, and imaging findings after three admissions, he was diagnosed with IHCs. This case demonstrates the challenges in diagnosing IHCs in patients with multiple hepatic cysts and highlights the necessity of a careful follow-up of clinical histories and findings of definitive imaging tests in the diagnosis of IHCs in patients with recurrent fever. To diagnose IHCs effectively, a comprehensive approach including history taking, physical examination, and diagnostic testing, is essential. IHCs should be considered by physicians when patients present with recurrent fever. To avoid missing IHCs, physicians in outpatient departments should continuously follow up on patients’ IHC-related symptoms such as fever and right upper quadrant pain.

## Introduction

Diagnosing infectious hepatic cysts (IHCs) can be challenging, and patients with IHCs may present with various symptoms. The typical symptoms include fever and abdominal pain; however, there could be other symptoms depending on the location of the infection [1]. Previous studies have shown that infection of liver cysts in the central part of the liver can cause vague symptoms, such as fever and fatigue [1,2]. Superficial infectious liver cysts can cause abdominal pain [3]. Furthermore, when patients have multiple liver cysts and infections, identification of the location of infection among the cysts can be challenging [1-3].

The three treatment options for IHCs are antibiotics, transcutaneous interventions, and surgery [4]. Previous studies have shown various durations of antibiotic treatment, ranging from two to eight weeks. As the rate of completion of antibiotic treatment is approximately 50%, many patients also have to undergo percutaneous interventions or surgery [2,4]. For these latter treatment options, the detection of infectious parts in the liver is essential. However, diagnosing and determining the treatment course of IHCs can be challenging in patients with co-existing polycystic liver disease.

We encountered a case where a patient presented with a fever of unknown origin and was finally diagnosed with an infection of polycystic liver cysts based on imaging and bacterial tests. We present this case to demonstrate the challenges of making a diagnosis in cases of IHC.

## Case presentation

A 70-year-old man visited a rural community hospital with a chief complaint of recurrent fever and pain in the right upper quadrant. One year prior, he had presented with fever and right pleural pain at a university hospital where he was diagnosed with pleuritis without any positive bacterial culture. His past medical history included hypertension, dyslipidemia, lacunar infarction, autosomal dominant polycystic kidney disease (ADPKD) with polycystic liver disease, benign prostatic hyperplasia, and bacterial pleuritis; however, the patient was in good health until two months prior to the current presentation. His medications included cilostazol, febuxostat, atorvastatin, telmisartan, amlodipine, nifedipine, and tamsulosin. 

The patient’s vital signs were as follows: blood temperature, 38.2 °C; blood pressure, 149/77 mmHg; heart rate, 97 beats per minute; respiratory rate, 18 breaths per minute; and SpO2, 93%. Physical examination did not reveal any enlarged cervical lymph nodes. Inspiratory late crackles were heard in the right lower lung. The abdomen was soft with flat tenderness in the right upper quadrant and indirect fist percussion of the liver without Murphy’s sign. There was no evidence of arthritis in the knees or hands. Laboratory data showed no abnormality in liver function with elevated C-reactive protein levels (Table 1).

**Table 1:** Laboratory data two months prior and on the day of admission Na: Sodium; K: Potassium; Cl: Chlorine; IgG4: Immunoglobulin G4

Markers	Two months prior	Day of admission	Ranges
White blood cells	14.1 × 10^3^/μL	8.3× 10^3^/μL	3.5–9.1 × 10^3^/μL
Red blood cells	4.11 × 10^6 ^/μL	3.34 × 10^6 ^/μL	3.76–5.50 × 10^6^/μL
Platelets	10.0 × 10^4 ^/μL	17.3 × 10^4 ^/μL	13.0–36.9 × 10^4^/μL
Total protein	7.7 g/dL	6.4 g/dL	6.5–8.3 g/dL
Albumin	4.1 g/dL	2.7 g/dL	3.8–5.3 g/dL
Total bilirubin	1.5 mg/dL	0.6 mg/dL	0.2–1.2 mg/dL
Direct bilirubin	0.5 mg/dL	0.3 mg/dL	0–0.4 mg/dL
Aspartate aminotransferase	23 IU/L	25 IU/L	8–38 IU/L
Alanine aminotransferase	21 IU/L	26 IU/L	4–43 IU/L
Alkaline phosphatase	122 U/L	256 U/L	106–322 U/L
γ-Glutamyl transpeptidase	66 IU/L	106 IU/L	<48 IU/L
Blood urea nitrogen	17.6 mg/dL	34.1 mg/dL	8–20 mg/dL
Creatinine	1.26 mg/dL	1.61 mg/dL	0.40–1.10 mg/dL
Serum Na	133 mEq/L	132 mEq/L	135–150 mEq/L
Serum K	4.1 mEq/L	4.1 mEq/L	3.5–5.3 mEq/L
Serum Cl	96 mEq/L	102 mEq/L	98–110 mEq/L
C-reactive protein	14.5 mg/dL	13.9 mg/dL	<0.30 mg/dL
IgG4	-	81 mg/dL	<135 mg/dL
Urine test			
Leucocyte	(−)	(−)	-
Protein	(−)	(−)	-
Glucose	(−)	(−)	-
Bilirubin	(−)	(−)	-
Ketone	(−)	(−)	-
Blood	(−)	(−)	-
Antinuclear antibody	-	<40	-
Interferon-Gamma Release Assays	-	(−)	-

**Figure 1:**
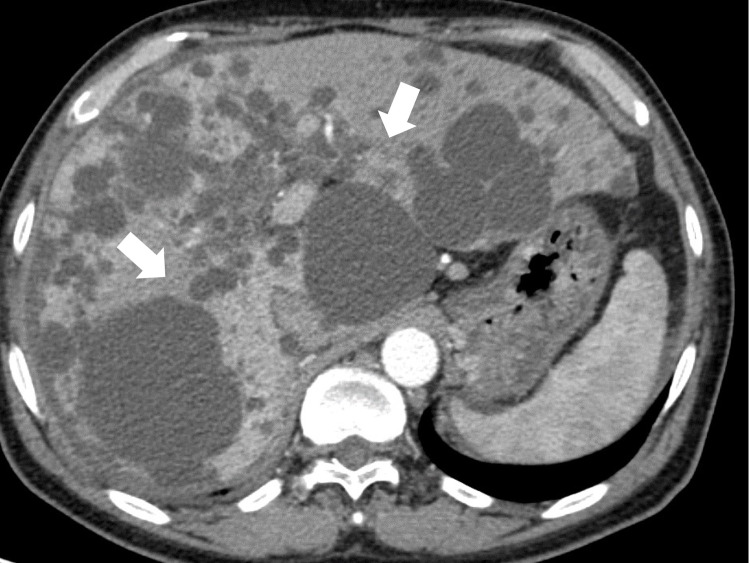
Enhanced computed tomography of the liver two months prior to the patient’s admission. Several cysts (white arrows) are visible in the liver; however, no infectious finding is detected.

Contrast-enhanced computed tomography (CT) of the chest, abdomen, and pelvis revealed no evidence of IHCs, any intra-abdominal abscess, or a solid renal or hepatic mass (Figure 1).

He was tentatively diagnosed with an IHC. Blood cultures were obtained, and the patient was treated with cefmetazole. After the treatment was initiated, his fever resolved. Since there was no evidence of bacteremia, a urinary tract infection, or IHCs, his treatment was discontinued on the 10th day and he was discharged. After discharge, the patient was followed up at the outpatient department for recurrent symptoms. 

**Figure 2:**
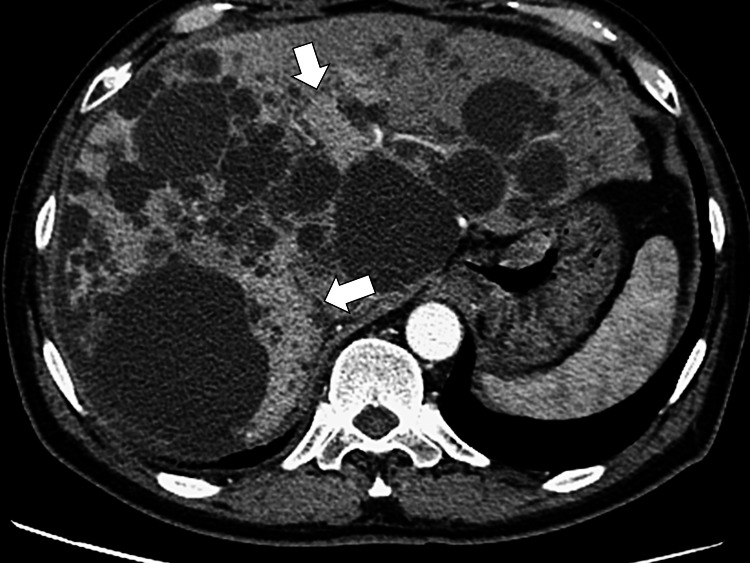
Enhanced computed tomography of the liver on admission. Enhanced areas around specific cysts indicate inflammation.

One day before his scheduled follow-up visit, he developed pain in the right upper quadrant and fever, for which he visited our hospital and was admitted. His vital signs were normal except for a fever of 38.6 °C. Physical examination revealed right pleural pain, late crackles on the right chest, and no abdominal tenderness. Laboratory data revealed no abnormalities in liver function (Table 1). Two days after admission, one set of blood cultures grew *Klebsiella pneumoniae*. We performed enhanced computed tomography (CT) of the liver to ascertain an infectious liver cyst. CT revealed an enhanced area on several cysts in the liver (Figure 2).

The patient was then diagnosed with bacteremia and IHCs; he was treated with cefotiam based on the culture results. The 14-day treatment was effective for this patient. He was then referred to the university hospital for further interventions for infectious liver cysts and the prevention of recurrent infections. The patient was diagnosed with an infection of multiple hepatic cysts at the university hospital. Considering the risk of operation of the cysts and recurrence of infections, he was treated with minocycline.

## Discussion

This case demonstrated the need for the persistent approach in diagnosing IHC in patients with multiple hepatic cysts and revealed that careful follow-up of patients' clinical histories and findings of definitive imaging tests can aid in the diagnosis of IHCs in patients with recurrent fever.

Diagnosing IHCs is challenging due to the difficulty in detecting the location of infections. IHCs are more often found in women than in men, with an estimated female-to-male ratio of 1-1.5:1 [1]. Symptoms include fever, chills (50%), and abdominal pain (41.7%) [4]. ADPKD is prevalent in patients with IHC [2]. In our case, the patient had ADPKD and multiple cysts with abdominal pain; therefore, CT could not detect the location of the infection as several lesions were enhanced in the liver [5,6]. In addition, there are various pathways for the infection of liver cysts. Therefore, a diagnosis of IHCs should be made based on a range of clinical information and considering the pathophysiology of the patient’s condition.

Blood cultures may be effective in the diagnosis of IHCs; however, they lead to successful pathogen isolation in only about half of IHC cases [7]. Furthermore, bacteria are isolated from aspirate cultures in only 52% of IHC cases. *Klebsiella pneumoniae* was revealed to be the most commonly isolated pathogen in a Japanese study [8]. An infection could be caused by the surrounding organs, hematogenous seeding from the systemic circulation, biliary infection, protein vein pyemia, or trauma [8]. In our case, there were no preceding events of trauma or liver function abnormalities that could lead to vein pyemia or transient infection from the biliary tract without biliary infections. Therefore, a comprehensive approach is required for the diagnosis of IHCs, including continuous history taking, physical examination, blood tests, and additional imaging tests.

Previous studies have shown that enhanced CT and magnetic resonance imaging (MRI) do not have high sensitivity and specificity; therefore, imaging tests alone cannot reliably diagnose IHCs [2,9,10]. In our case, the patient was not diagnosed during the first three admissions. For the diagnosis of causes of fever of unknown origin and recurrent fever, imaging tests can be used; however, their diagnostic accuracy may not be high. In addition, the combined use of CT and MRI may not improve the validity of IHC diagnosis [9,10]. The diagnosis of IHCs should thus be performed clinically, including history taking and physical examination.

A practical approach for the diagnosis of IHC should include follow-up of symptoms, consideration of the vagueness of symptoms in older patients, and imaging tests at appropriate intervals. Older patients tend not to have specific symptoms and do not seek help from others until the symptoms get severe [11,12]. Continuous follow-up without early closure as a diagnostic bias is essential to diagnose IHCs. Besides, older people, especially those living in rural areas, may prefer primary care physicians [13-15]. Therefore, primary care physicians living in rural areas should follow up on the patients’ symptoms (such as fever of unknown origin) persistently not to miss the diagnosis of IHCs. 

In this case, we followed up with the patient post-discharge to identify definitive diagnostic clues such as changes in MRI and CT or blood cultures. For the diagnosis of IHCs, follow-up of symptoms is critical because IHCs can recur in more than 20% of patients [8]. Without a clinical follow-up, patients may visit other medical institutions because of their changing help-seeking behaviors from rural to urban institutions, thus bypassing primary care [16,17]. Patients with a previous IHC should be followed up to detect symptoms related to the IHC to determine the optimal timing for interventional treatments.

## Conclusions

The diagnosis of an IHC can be often missed, and therefore needs persistence to be diagnosed. To diagnose IHC effectively, a comprehensive approach, including history taking, physical examination, and diagnostic tests, is essential. IHCs should be considered by physicians when patients present with recurrent fever. To avoid missing an IHC, primary care physicians should carefully follow up various symptoms in patients presenting in outpatient departments, while respecting their help-seeking behaviors.
